# Associations between subclinical bovine respiratory disease, growth patterns, and the nasal and fecal microbiota in dairy replacement heifers: a retrospective study

**DOI:** 10.3389/fvets.2026.1813534

**Published:** 2026-05-08

**Authors:** Sabine Scully, John Donlon, Bernadette Earley, Paul E. Smith, Michelle Stafford, Catherine McAloon, Christina Strube, David A. Kenny, Sinéad M. Waters

**Affiliations:** 1 Department of Animal Bioscience Research, Animal and Grassland Research and Innovation Center, Teagasc Grange, Dunsany, Ireland; 2 School of Veterinary Medicine, University College Dublin, Dublin, Ireland; 3 School of Biological, Earth and Environmental Sciences, University College Cork, Cork, Ireland; 4 Institute for Parasitology, Center for Infection Medicine, University of Veterinary Medicine Hannover, Hannover, Germany

**Keywords:** calf health, clinical respiratory scoring, metataxonomics, microbiota, replacement heifer, subclinical bovine respiratory disease, thoracic ultrasonography

## Abstract

Subclinical bovine respiratory disease (BRD) is difficult to detect using clinical signs alone, and its broader associations with growth and microbial communities remain poorly defined. This retrospective observational study classified twenty Holstein-Friesian and Jersey replacement heifers at weaning using clinical respiratory scoring and thoracic ultrasonography (TUS). Ten heifers had no clinical signs of BRD and no lung lesions, while ten showed no clinical signs but had ultrasonographic lung consolidation >1 cm in diameter. The study investigated (1) associations between subclinical BRD status at weaning and liveweight patterns from birth to early gestation, and (2) the nasal and fecal microbiota associated with BRD status at weaning and again at 8 mo of age. Heifers were clinically assessed from birth to 8 mo, with TUS, nasal swabs and fecal samples collected at weaning and 8 mo. Liveweights were recorded from birth to early gestation. Microbial community composition of nasal and fecal samples from weaning and 8 mowere characterized by 16S rRNA gene amplicon sequencing. Across the study period, heifers classified as healthy were heavier and had greater average daily gains than those with subclinical BRD. BRD status was associated with nasal microbiota composition at weaning and showed a tendency in fecal samples at weaning. No differences were detected at 8 mo. Dominant nasal genera included *Moraxella, Pasteurella, Mannheimia* and *Mesomycoplasma*, none of which differed by health status. Nine nasal and 24 fecal amplicon sequence variants (ASVs) were associated with BRD-status, although all were low abundance taxa (relative abundance < 1.5%). *Romboutsia ilealis* (nasal) was consistently more abundant in healthy heifers, while *Butyricioccaceae* UCG*-*009 (fecal) was more abundant in the subclinical group at both timepoints. These findings highlight associations between subclinical BRD detected at weaning and both growth patterns and specific low abundance taxa. Further studies with larger cohorts are required to clarify these relationships and their biological relevance.

## 1 Introduction

Bovine respiratory disease (BRD) is a major cause of morbidity and mortality in young stock across all production systems (1). In Ireland BRD is a leading cause of cattle death, particularly in calves (2). The many impacts of BRD pose significant challenges to animal health and welfare and compromises the sustainability of dairy production systems (3). The prevalence of BRD varies across Irish dairy farms, with median within herd prevalence ranging from 0 to 22% (4). Regardless, respiratory infection remains the primary cause of death in calves one month of age and older, with mortality rates in 2023 reported as 39% of all post-mortem laboratory submissions in one to six months old calves and 41% in calves of six to 12 months old (2). The term BRD encompasses both upper (URT) and lower respiratory tract (LRT) infections (5). Preventing and managing BRD is challenging because respiratory disease is multifactorial and often the result of co-infection, where a primary viral infection predisposes the animal to secondary bacterial infection (6, 7). The risk of BRD is associated with multiple factors including microbial exposure, host immunity, environmental conditions and animal management practices (8–11). A recent meta-analysis by Buczinski et al. (3) on the effects of BRD, found that early-life BRD was associated with increased risk of death, higher likelihood of culling before first calving, reduced average daily gains and reduced milk yields during first lactation. Animals with a history of BRD during early life have also been found to have poorer fertility (12, 13). These consequences result in serious economic losses (5), as well as decreased welfare and may predispose the animal to future negative health events. Current recommendations for the control of BRD emphasize prevention through supporting optimal immunological development in calves (high-quality colostrum, adequate nutrition, vaccination etc.), implementation of appropriate biosecurity, housing and management practices (14) and early detection of disease (4, 15).

Early detection of BRD is critical for effective disease management and requires a combination of diagnostic tools. This includes the use of clinical assessment and auscultation as well as more advanced technologies such as thoracic ultrasonography (TUS). Clinical assessment scoring (CAS) uses observation of clinical signs of disease and attributes a score to the severity of the observation. Commonly used systems include the Wisconsin-Madison system (16) and the California system (17). However, recent research indicates that reliability of these scoring systems is highly dependent on score interpretation and can vary widely (18). They also rely on the presence of observable clinical signs, making their use in early detection of subclinical disease ineffective. TUS allows for the visualization of lung consolidation and its severity prior to the presentation of clinical signs (5, 19, 20), indicating that it is a promising tool for early detection. Combining the use of CAS with TUS, bovine respiratory disease can be classified into three subtypes: Upper respiratory infection: CAS positive and TUS negative (without lung lesions); Clinical BRD: CAS positive and TUS positive (presence of lung lesions); Subclinical BRD: CAS negative and TUS positive (presence of lung lesions) (4, 15, 21).

The URT and LRT microbiomes are of increasing interest in BRD research, particularly their role in disease manifestation (22). Microbiomes at the mucosal surface, consisting of microorganisms, their by-products, environmental and host-derived components, play an essential role in health maintenance of the animal (23). The use of Next Generation Sequencing has shown that the bovine nasal microbiota of beef calves resembles that of the cow from birth to weaning; however, after weaning, the abundance of BRD-associated bacteria such as *Mannheimia, Pasteurella*, and *Mycoplasma* increased (24). In dairy calves, Lima et al. (25) established that the calf URT, regardless of age, was very similar in microbial composition to that of the dam vaginal microbiota. They also reported that *Mannheimia, Moraxella, Bacteroides, Streptococcus* and *Pseudomonas* were the most proportionally abundant genera in the calf URT (25). Both Uddin et al. (24) and Lima et al. (25) concluded that *Mannheimia* in the calf URT originated from the dam's vaginal microbiota. Common BRD-associated pathogens have also been observed in the nasal microbiota of feedlot and beef cattle with and without BRD (26–28). Other studies, performed in dairy calves, have reported the presence of BRD-associated pathogens in the nasal microbiota in varying abundances in both diseased and non-diseased animals (29–31). Recent work has shown that the prevalence of these BRD-associated bacteria was not associated with disease status (32, 33). The presence of these bacteria in both healthy and diseased animals throughout the literature supports the assertion that, while opportunistically pathogenic, they are normal members of the URT microbiota (22, 23, 34). Disease manifestation is often linked to dysbiosis of the microbial community (22, 23). Recent research has emphasized the importance of not only focusing on dominant microbiota, but also on those that are low in abundance or rare, as these are more sensitive to disturbances and may play key roles in immune development and disease outcomes (35).

Research studies in human medicine have shown that viral respiratory infection can alter the gut microbiota and the mucosal epithelial barrier (36). Additional studies have shown that dysbiosis of the gut microbiota increases the risk of lung infections (37) and that gut probiotic administration has potential for preventing and managing respiratory disease (38). These findings support the concept of the microbiome-gut-lung axis which highlights the bidirectional relationship between respiratory function, gut health and microbiota composition (39). Due to the asymptomatic nature of subclinical disease, it remains challenging for investigation, resulting in a limited understanding of its long-term effects in replacement heifers. Subclinical BRD may influence the composition of the nasal microbiota in affected calves; however, to the authors' knowledge, no studies have investigated the relationship between subclinical BRD and the hindgut microbiota. Furthermore, there is a lack of research examining nasal microbiota in relation to naturally occurring BRD within pasture-based production systems. Therefore, the objectives of this study were to (1) explore associations between subclinical BRD and growth patterns of dairy replacement heifers; (2) identify microorganisms in the nasal and fecal microbiota associated with health status at weaning and (3) explore associations between subclinical BRD, the nasal and fecal microbiota following initial disease detection and pasture turnout.

## 2 Materials and methods

### 2.1 Ethics statement

The experiment was undertaken at the Dairygold Research Farm in Kilworth, Co. Cork, Ireland (Teagasc, Animal and Grassland Research and Innovation Center, Moorepark, Fermoy, Co. Cork, Ireland; 52°09′N; 8°16′W). All animal procedures performed were undertaken by trained research personnel, were approved by the Teagasc Animal Ethics Committee, and are consistent with the experimental license (AE19132/P148) issued by the Irish Health Products Regulatory Authority in accordance with European Union legislation (Directive 2010/63/EU) for the protection of animals used for scientific purposes.

### 2.2 Animal management

**Figure 1:**
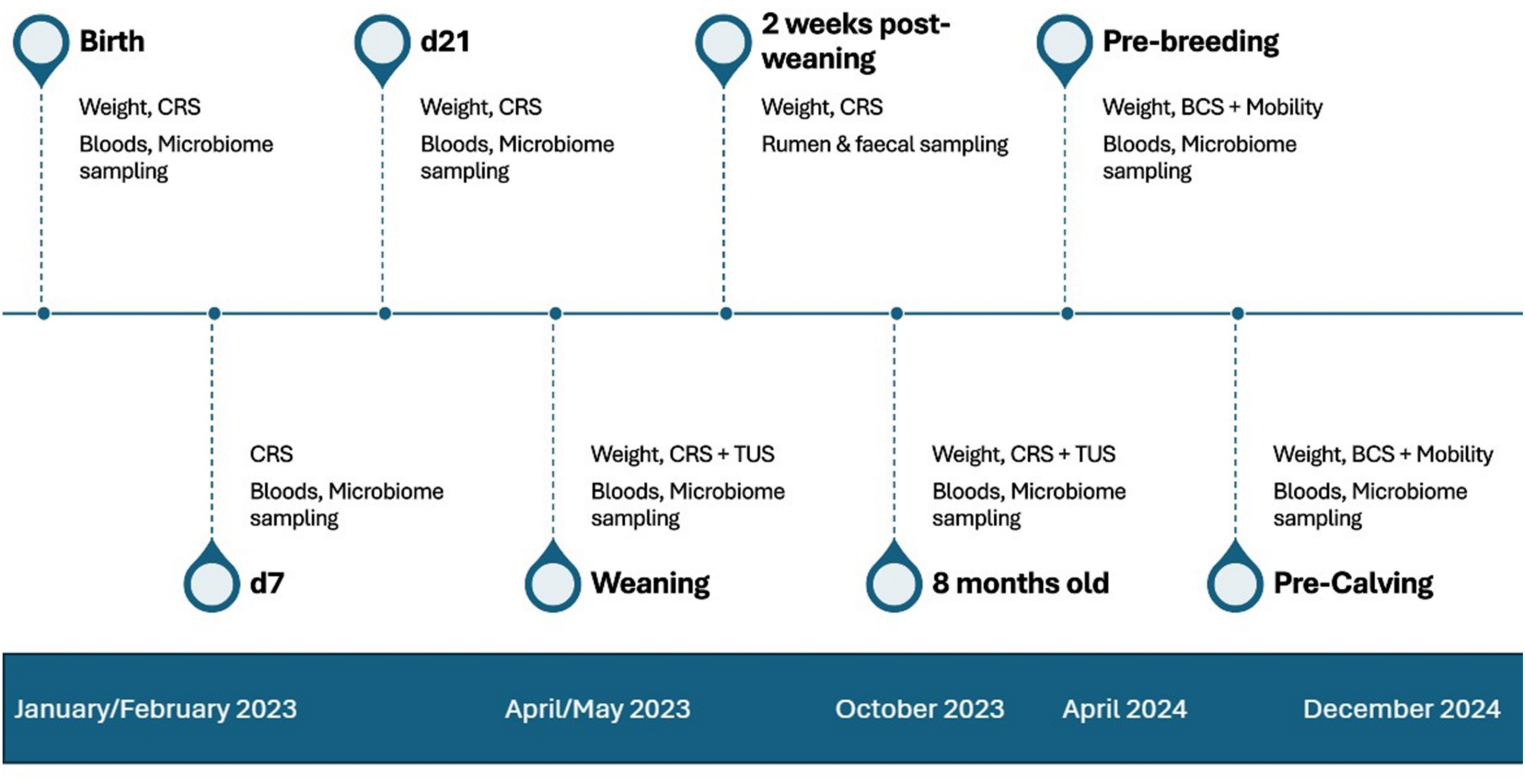
Trial work and sample collection timeline from start of calving in January 2023 to pre-calving in December 2024.

During the 2023 spring calving season (January to March) a total of 60 Holstein-Friesian and Jersey heifer calves were enrolled in a larger longitudinal study investigating bovine microbiome development. Calves were clinically assessed using a modified Wisconsin-Madison calf health scoring system [available on Open Science Framework (OSF)] (40) across multiple time-points from birth to 8 mo (birth, d 7, d 21, weaning, 2 weeks post-weaning, 8 mo). Alongside clinical assessment, calves underwent TUS imaging at weaning [mean age d90 (SE 0.31)] and 8 mo mean age d244 (SE 1.90), where scores were determined using methodology previously described by Donlon et al. (4). Heifer calves were periodically sampled across various body sites from birth to first calving (Figure 1). Weights were collected at each sampling timepoint. Weights collected at birth and weaning were used to calculating pre-weaning average daily gain (ADG), weaning and 8 mo were used to calculate post-weaning ADG, and weights collected at 8 mo and two months after first insemination service were used to calculate post-service ADG. These were calculated by subtracting weight 1 from weight 2 and dividing by the number of days in between the two timepoints as depicted in Equation 1. This is the standard formula for calculating ADG.

Equation 1 Standard formula for calculating Average Daily Gain (ADG) of dairy heifer calves over a particular period of time


$$\begin{array}{l} Average\;Daily\;Gain=\frac{\left(weight\;2-weight\;1\right)}{\left(days\;old\;at\;date\;2-days\;old\;at\;date\;1\right)} \end{array}$$

Clinical assessments were performed by Scully S, Donlon J, and Stafford M, where initially scoring was performed together to ensure consistency across observers and time-points. TUS was performed by Donlon J and Scully S. All scores were cross validated between scorers. Based on clinical respiratory scores (CRS) and TUS scores recorded at weaning, a cohort of 20 replacement heifers were retrospectively selected to investigate the effect of subclinical BRD on long-term growth performance and the nasal and fecal microbiota.

#### 2.2.1 Birth to weaning

All calves were managed under identical conditions, following the standard operating procedures of the Dairygold research farm (Teagasc Moorepark, Fermoy, Co. Cork, Ireland). Dam and calf management have been previously detailed by Scully et al. (40, 41). Briefly, calves were separated from the dam within 30 min of birth, weighed using a calibrated scale, and had their navels disinfected with Foradine 10 (10% povidone iodine). Within 2 h of birth, calves received 8.5% of their birth weight in liters of colostrum [mean volume of 2.68 L (SE 0.03)], fed manually, either by bottle and teat or esophageal tube. Colostrum was sourced from the maternal herd and stored by individual donor, in a refrigerator (4°C) for no more than 24 h. Colostrum was combined with that of a second donor, when necessary, to ensure the calf received the appropriate volume. Colostrum was re-heated in a water bath to 38°C for 60 min prior to being administered to the calf. Colostrum quality was assessed in field, prior to being fed to the calf, using a BRIX refractometer and later subjected to single radial immunodiffusion (sRID) for the quantification of total immunoglobulin (Ig) G concentrations. Using a fresh sRID kit, the same methodology described by Scully et al. (41) was performed on colostrum to quantify total colostral Ig G concentrations, however, samples were diluted using a different ratio (1:5 or 1:6; additional data available on OSF). Calves were then housed individually in deep-bedded straw pens (1.4 m × 0.46 m) until d 3 post-birth. From d 3 post-birth to weaning, calves were housed in group pens (9.1 m × 4.6 m) with deep straw bedding and access to outdoor runs in groups of 40 calves of similar age. From second feed to d 2 post-birth calves were fed 4 L transition milk/calf per day via bucket and teat. From d 3 to d 14 post-birth calves were fed transition milk via trough and teat, twice daily, gradually increasing from 4 L to 6 L/calf per day. From d 14 post-birth onwards, calves were fed 6 L of milk replacer (Heiferlac GB/IRL, Volac Feeds LTD, Ireland; 26% crude protein) per calf per day via trough and teat. From d 3 onwards, calves had free access to water, roughage (straw), and pelleted concentrates (Sweet start calf starter pencils, Southern Milling LTD, Cork, Ireland; 20% crude protein). From second feed, for 7 days, calves received paromomycin (Parafor™, BIOVET JSC, Bulgaria) as a preventative against cryptosporidiosis. At two weeks post-birth, heifers were dosed with toltrazuril (Bovicox, KRKA, Slovenia) for the control of coccidiosis and vaccinated against respiratory diseases (Pneumovac PLUS, Animal Health Distributors, Ireland). At four weeks post-birth calves were vaccinated against *Clostridia* (Tribovax10, Intervet Ireland LTD., Dublin, Ireland). Weaning was gradual over seven days, based on breed-specific target weights (Holstein-Friesian: 85 kg; Jersey 75 kg) and concentrate intake (minimum of 1 kg/calf for 3 consecutive days).

#### 2.2.2 Post-weaning to first service

After weaning, calves were turned out to pasture and offered 1 kg of concentrates/heifer per day (Super calf rearer nuts, Southern Milling LTD., Cork, Ireland; 17% crude protein) until first housing in November 2023. Weanlings were vaccinated against Infectious Bovine Rhinotracheitis (IBR; Rispoval^®^ IBR-Marker inactivated, Zoetis Belgium S.A., Dublin, Ireland) at 6 mo and received booster vaccines at 12 mo. After pasture turnout, heifers were regularly assessed for endoparasites using fecal egg counts. Heifers received a 25 mL dose of albendazole (Albex^®^ 10% w/v; Chanelle Animal Health LTD., Ireland) orally at the end of August 2023, and again at the end of September 2023. All heifers were moved indoors for winter housing in November 2023. From this point on heifers received no concentrate supplementation and were fed grass silage *ad libitum*. At 14 mo, heifers were vaccinated against Bovine Viral Diarrhea Virus (BVD, Bovilis^®^BVD, Intervet Ireland LTD., Dublin, Ireland) and leptospirosis (Bovilis^®^ Leptavoid^®^-H, Intervet Ireland LTD., Dublin, Ireland). Heifers were artificially inseminated at the end of April 2024. After first service, heifers were turned out to pasture with a stock bull until November 2024. No concentrate supplementation occurred at pasture. At 20 mo, heifers were vaccinated against salmonellosis (Bovilis^®^ Bovivac S, Intervet Ireland LTD., Dublin, Ireland).

### 2.3 Clinical assessment and thoracic ultrasonography

#### 2.3.1 Clinical assessment scoring

Calves were clinically assessed prior to sample collection, starting with the least to most invasive (rectal temperature) in a cranial-caudal fashion. Clinical assessment was performed prior to TUS and included the Wisconsin-Madison calf health scoring system (16), rectal temperature, skin-tent test for dehydration, coat condition and scoring of the mucus membranes (42). The Wisconsin-Madison calf health scoring system scores on a scale of zero to three, where zero is normal and three is severely abnormal. The scoring system includes assessment of nasal discharge, ocular discharge, ear position/head tilt, cough, feces, naval and joints. CRS was determined by combining nasal discharge, ocular discharge, ear position/head tilt and cough scores. A CRS of three or greater was determined to be indicative of clinical BRD, a more aggressive threshold than that put forward by McGuirk and Peek (16), where the authors propose that a CRS ≥5 is indicative of clinical BRD.

#### 2.3.2 Thoracic ultrasonography scoring

Thoracic ultrasonography protocol has been described in detail by Donlon et al. (4). Briefly, TUS was performed with a portable linear rectal ultrasound scanner set at a depth of 8 cm and a frequency of 8.0 MHz (Tecnoscan SR-1 C, Improvet, Spain.). Similar to the method by Ollivett and Buczsinski (43), calves were scanned starting at the 10th intercostal space (ICS) moving to the first ICS on the right side and then from the 10th ICS moving to the second ICS on the left side. Calves were scored on a scale of 1–5 where scores of 0 and 1 are considered normal, 2a indicates the presence of lung lesions < 1cm in diameter in a single lobe, 2b indicates the presence of lung lesions ≥ 1cm in diameter in a single lobe, score 3 indicates the presence of full thickness consolidation in only one lobe, score 4 indicates full thickness consolidation in two lobes and a score 5 indicates full thickness consolidation in three or more lobes (4, 43). Calves were scanned at weaning and again at 8 mo.

#### 2.3.3 Selection criteria

Clinical assessment and TUS records for all heifers from birth to 8 mo were compiled. For the present study, animals were retrospectively selected based on their initial TUS scores and clinical assessment findings at weaning. Heifers were considered healthy if they had a cumulative (ear, nasal, ocular and cough) CRS of < 3 at weaning and a TUS score of ≤ 1 (Data available on OSF). Heifers were determined to have subclinical respiratory disease if they had a cumulative CRS of < 3 and a TUS score ≥ 2B, indicating the presence of lung lesions ≥ 1cm in diameter. A total of 20 age-matched Holstein-Friesian (*n* = 14) and Jersey (*n* = 6) heifers, 10 healthy and 10 subclinical, were selected for inclusion (Table 1). Nasal swab and fecal samples from these heifers, collected at weaning (d 90) and 8 mo (d 244), were used for microbiota analysis.

**Table 1:** Sample population by calf breed and calf health status at weaning.

Sample pool frequencies			
	Healthy (*n* = 10)	Subclinical BRD (*n* = 10)	Total (*n* = 20)
Holstein-Friesian (*n* = 14)	7	7	14
Jersey (*n* = 6)	3	3	6
Total (*n* = 20)	10	10	20

### 2.4 Sample collection and processing

All sample collection was performed while wearing clean nitrile gloves sterilized with 70% ethanol/molecular water solution (EMS). Samples were collected in a cranial-caudal direction. All laboratory procedures were performed in a Class II biological safety cabinet fitted with a HEPA filter (MSC-Advantage, Thermo Scientific). Sample processing order was determined on a random basis, and all workspaces and equipment were then cleaned, disinfected and sterilized. Nasal swab processing, microbial DNA extraction, purification and qualitative polymerase chain reaction (qPCR) were performed first. All workspaces and equipment were again cleaned, disinfected and sterilized. Processing, microbial DNA extraction, purification and qPCR of fecal samples were performed next. One nasal swab and two fecal samples at weaning and one fecal sample at 8 mo were not located.

#### 2.4.1 Nasal swab collection and sample preparation

Nasal swabs were collected using Puritan^®^ PurFlock Ultra^®^ Sterile Flocked Collection Devices (Puritan Medical products Company LLC., Maine, USA). Similar to the protocol described by Esnault et al. (44), the outer nose of the calf was wiped clean with a clean paper towel sprayed with 70% EMS when necessary (i.e., dirty nose, excessive debris). Two nasal swabs were collected per nostril, totaling four nasal swabs per calf. Packaging for each nasal swab was opened and removed by the base of the swab immediately prior to insertion into the nasal cavity. The flocked end of the swab was inserted approximately 14 cm into the nostril and spun gently in a circular motion for approximately 10 s. The swab was then carefully removed, ensuring no contact with any other surfaces or materials and the tip was placed immediately in a sterile 2 mL Eppendorf tube. The swab tip was cut with clean, sterilized (70% EMS) scissors prior to closing the Eppendorf. Swabs were then snap frozen in liquid nitrogen, stored on dry ice and transported to the laboratory and stored at −80°C until further analysis. Two swabs from each animal for each time-point (weaning and 8 mo) were selected for microbial DNA extraction. Swabs were removed from −80°C and kept on ice during processing. One mL of molecular biology-grade phosphate-buffered saline (PBS; Invitrogen™ Phosphate-buffered saline (10X) pH 7.4, RNase-free; Ambion™, Applied BioSystems) was added to each swab-containing Eppendorf, which was then vortexed for 1 min. The swab was then removed from the PBS wash, using sterile, single-use forceps (Buerkle™ disposable forceps, Fisher Scientific), and combined with the 1 mL PBS wash from the second swab collected from the same animal to form 2 mL of PBS wash for microbial DNA extraction. From this 250 μL of nasal swab wash was used for microbial DNA extraction.

#### 2.4.2 Fecal sample collection and sample preparation

Voided fecal samples were collected where possible. When natural defecation did not occur, samples were collected via rectal stimulation. Samples were placed in a sterile 25 mL screw cap tube (Sarstedt AG & Co, Germany) and immediately snap frozen in liquid nitrogen. Samples were then stored on dry ice and transferred to the laboratory and stored at −80°C until further analysis. Prior to microbial DNA extraction, samples were processed as described by Scully et al. (40). Briefly, fecal samples were ground into a fine powder, under liquid nitrogen, using a pestle and mortar. For microbial DNA extraction, 250 mg of ground feces were used.

#### 2.4.3 Blood sample collection and sample preparation

Whole blood samples collected at d 7 post-birth, weaning, and 8 mo were used to quantify Ig G concentrations (serum; d 7), haptoglobin concentrations (plasma; weaning and 8 mo) and anti-*Dictyocaulus viviparus* antibodies by ELISA (serum; 8 mo). Whole blood for serum was collected via jugular venipuncture using an 18 g × 1” needle (Vacutainer^®^ PrecisionGlide™ Single Sample Veterinary Needle, Becton Dickinson) inserted into a 1 × 9 mL VACUETTE^®^TUBE Serum Clot Activator (Greiner Bio-One, Austria). Whole blood for plasma was collected in the same manner as serum, using a 1 × 9 mL VACUETTE^®^ Lithium Heparin tube (Greiner Bio-One, Austria). After collection, whole blood samples were left at room temperature for approximately 90 min and then refrigerated at 4°C for no more than 12 h. Whole blood for serum was centrifuged at 1,600 × g for 10 min at 20°C. Serum was then collected and aliquots (3 × 1 mL) were transferred to sterile Eppendorf tubes. Whole blood for plasma was centrifuged at 1,600 × g for 15 min at 20°C. Plasma was then collected and aliquots (3 × 1 mL) were transferred to sterile Eppendorf tubes. Both serum and plasma were stored at −80°C until further analysis.

### 2.5 Blood tests

Passive transfer of immunity (PTI) testing was performed by quantifying serum total protein (STP) using digital refractometry and serum total IgG (sIgG) concentrations via single radial immunodiffusion (sRID) assay. Serum samples collected on d 7 were thawed for approximately 24 h by moving samples from −80°C to +4°C. Samples were then removed from the refrigerator and allowed to come to room temperature (20°C) for 1 h. Serum total protein and sRID methodologies have been previously detailed by Scully et al. (40). Additionally, colostrum fed to calves was subjected to sRID analysis for the quantification of total colostral Ig G concentrations, using the same methodology as Scully et al. (41). Heifer PTI status was defined using the recommendations of Lombard et al. (45): excellent (STP: ≥ 6.2 g/dL; sIgG: ≥ 25.0 mg/mL); good (STP: 5.8–6.1 g/dL; sIgG: 18–24.9 mg/mL); fair (STP: 5.1–5.7 g/dL; sIgG: 10–17.9 mg/mL); poor (STP: < 5.1 g/dL; sIgG: < 10 mg/mL).

For the quantification of haptoglobin, plasma samples collected at weaning and 8 mo of age were moved from −80°C to −20°C approximately one week prior to analysis. Samples were moved from −20°C to +4°C the day prior to laboratory analysis. Haptoglobin assays were carried out using a commercially available kit (TP-801, Tridelta Development LTD, Maynooth, Co. Kildare, Ireland) on an AU480 auto-analyzer (Beckman Coulter, Inc., California, USA) similar to the method described by Earley et al. (46). Based on recommendations put forward by Schmitt and Staufenbiel (47), normal bovine haptoglobin concentrations were considered normal if they fell within the range of 0.00–0.05 mg/mL as per assay manufacturers guidelines (Available on OSF). To account for potential lungworm exposure, *D. viviparus*-specific ELISA antibody testing was performed on serum samples collected at 8 mo using the method described by von Holtum et al. (48). Animals were considered seropositive for anti-lungworm antibodies if the optical density ratio (ODR) was ≥0.50.

### 2.6 Microbial DNA extraction, qPCR and sequencing

Microbial DNA was extracted from the nasal swab PBS wash and the ground feces via repeated bead-beating and silica spin column purification using the Qiagen DNeasy^®^ PowerSoil^®^ Pro Kit (Qiagen, Manchester, England, United Kingdom) based on protocols described by Yu and Morrison (49). Extractions were performed on nasal swab PBS wash, in batches of 12, which consisted of 10 samples and one blank swab negative control (i.e., an extraction performed on PBS wash from two sterile unused swabs; *n* = 4) and one negative extraction control (i.e., an extraction performed without any sample; *n* = 4). Nasal swab microbial DNA extractions were then subjected to DNA purification using the Genomic DNA Clean & Concentrator^®^-10 kit (Zymo Research Corp., Irvine, California, USA). Fecal sample extractions were performed in 12 batches, which consisted of 11 samples and one negative extraction control (*n* = 4). Fecal sample microbial DNA extractions were subjected to DNA purification using the same type of kit as the nasal swabs. To monitor DNA extraction efficiency, three DNA extractions from each extraction kit were performed on ZymoBIOMICS™ Microbial Community Standard (Zymo Research Corp., Irvine, California, USA). Concentrations and purity were quantified on the Nanodrop 1,000 spectrophotometer (Thermo Fisher Scientific), and high molecular weight DNA integrity was assessed by electrophoresis on 1.0% agarose gels.

Post-purification, all extracted DNA samples underwent 16S qPCR analysis to verify the presence of bacterial DNA prior to sequencing, as previously described by Scully et al. (41), following the methodology originally detailed by Kittleman et al. (50). The qPCR reaction was performed on an ABI7500 FAST qPCR machine (Applied Biosystems, UK) with 7500 Fast Software v2.3. Reaction conditions were 95°C for 20 s, then 40 cycles of 95°C for 3 s and 60°C for 30 s. After completion of the qPCR run, the cycle threshold was set to 0.2 and baseline was set to automatic then CQ values were calculated. Results from qPCR indicated sufficient levels of bacterial DNA present in extractions to proceed with sequencing.

Microbial DNA extractions were subjected to 16S rRNA gene amplicon library preparation and sequencing at a commercial laboratory (Macrogen, Seoul, South Korea). Samples underwent the same sequencing protocols detailed by Scully et al. (40, 41). In brief, microbial DNA extractions were subjected to two rounds of PCR amplification where the first round of PCR amplification, targeting the V4 hypervariable region of the 16S rRNA gene, was performed using the 515F/806R primers (51) designed with Nextera overhang adapters and Herculase II Fusion DNA Polymerase (Agilent, Santa Clara, CA, USA). Cycle conditions were as follows: 95°C for 3 min, 25 cycles at 95°C for 30 s, 55°C for 30 s, 72°C for 30 s, and then 72°C for 5 min. PCR amplicon purification was performed using the standard AMpure paramagnetic bead protocol (Beckman Coulter, Indianapolis, IN, USA). Following purification, amplicons were subjected to a second round of PCR to permit the attachment of dual indices and Illumina sequencing adapters using the Nextera XT indexing kit (Illumina, San Diego, CA, United States). Cycle conditions for the second round of PCR were 95°C for 3 min, 10 cycles at 95°C for 30 s, 55°C for 30 s, 72°C for 30 s and then 72°C for 5 min followed by an additional PCR purification with the AMpure paramagnetic bead protocol (Beckman Coulter, Indianapolis, IN, USA). Amplicons were pooled together in equal concentration, and subject to sequencing on the Illumina MiSeq using the 500-cycle version 2 MiSeq reagent kit (Illumina, San Diego, CA, USA) on one lane.

### 2.7 Sequencing analysis

Raw amplicon sequencing data were analyzed together in *R* (v. 4.3.3) using *DADA2* (v. 1.30.0) and submitted to the pipeline as described by Callahan et al. (52) with minor modifications as outlined by Smith et al. (53). Quality checks of both forward and reverse reads were determined based on the visualization of Q scores with the aim to ensure mean Q scores of > 30 were upheld for forward and reverse reads. Filtering and trimming of poor-quality reads and removal of primer sequences was conducted using the trimLeft function in *DADA2*. Identical sequences were combined using the de-replication function followed by the merging of forward and reverse reads. An Amplicon Sequence Variant (ASV) table was then constructed following which chimeric sequences were removed. Taxonomy was assigned at the species level to sequence variants using the SILVA database (v. 138.2) (54). A bootstrapping threshold of 80 was applied for taxonomic classification by incorporating minBoot = 80 as part of the assignTaxonomy function as described by Smith et al. (53). Sample metadata, sequence taxonomy, and ASVs were combined into a phyloseq object using *phyloseq* (v. 1.46.0) (55). Based on the plateauing of the generated rarefaction curve, sequencing was deemed to be conducted to a sufficient depth, and no rarefaction was performed. Following this, bacterial and archaeal ASVs, classified beyond the phylum level, were obtained. Using samples and negative controls, contaminants were identified and removed using a frequency- and prevalence-based approach with a threshold of 0.5 via *decontam* (v. 1.22.0) (56). Data was then separated by sample type (nasal or fecal) and time-point (weaning or 8 mo). Subsequently, alpha (α; Shannon Index for richness and evenness) diversity was calculated for each sample. For comparisons of beta (β; composition) diversity and differential abundance analysis, the relative abundance (RA) of ASVs were calculated and those which were not present in >0.01% were removed prior to further analysis. Beta diversity was depicted graphically using non-metric multidimensional scaling (NMDS) using Bray-Curtis plots.

### 2.8 Statistical analysis

Prior to animal selection for this study, experts within the HoloRuminant consortium were consulted regarding the minimum sample size necessary per treatment group (healthy or subclinical BRD) for microbiome analysis. Through these consultations and a power analysis for an experiment examining proportional differences between samples the sample size used in this study was found to be appropriately powered for microbiome analysis. Regardless, small sample size, mild subclinical BRD manifestation, lack of variation in disease severity and sampling at discrete timepoints means that results should be interpreted cautiously and cannot be used to infer causation.

#### 2.8.1 Immunological and performance data

All performance and immunological data were analyzed in *R* (v. 4.5.0) using *RStudio* (v. 2023.12.1+402). Data was checked for normality and homogeneity of variance using histograms, Q-Q plots, and formal statistical tests. Data was subjected to repeated measures ANOVA with fixed effects of health status and breed and their interactions. Differences between treatments were determined using the estimated marginal means (emmeans) package. Mean values were considered statistically significant at *P* ≤ 0.05. Non-significant terms (*P* > 0.05) were excluded from the final model.

#### 2.8.2 Sequencing data

Prior to assessing the effect of health status, breed and interactions on overall prokaryotic community structure, the homogeneity of group dispersions was assessed. Following this, PERMANOVA based on Bray-Curtis dissimilarities, 9,999 permutations and a significance level of P < 0.05 were implemented to determine if health status or breed affected prokaryotic community structure. Both assessment of homogeneity of group dispersions and PERMANOVA were conducted using *R* package *Vegan* (v. 2.6.6) (57), implemented through *Microbiome* (v. 1.23.1) (58). Sequencing data was separated by sample type and time-point for further analysis. Alpha (α)-diversity was analyzed by calculating Shannon Index scores through *Microbiome*, and Shannon Index scores were subjected to ANOVA in *R*. For differential abundance analysis, datasets were combined based on sample type, and analysis was performed at the species level. Differential abundance analysis of ASVs was performed using *MaAsLin2* (v. 1.22.0) (59) with time-point and health status as fixed effects. Breed, STP, and lungworm ELISA results were included as random effects and the Benjamini-Hochberg procedure was implemented to correct for false discovery rate [q value; adjusted p (adj.p)]. Spearman's rank-order correlation for non-parametric data was performed to explore potential associations between heifer growth, immunological measures, TUS scores and ASVs associated with BRD-Status (those ASVs with an adj.*p* value of ≤ 0.10). Correlations were run using the *rcorr* function in *Hmisc* (v. 5.2.3) in *RStudio* (v. 4.3.0). Correlations (effect size and strength of the correlation, denoted as r_s_) were described using the following: 0.00–0.19, “very weak”; 0.20–0.39, “weak”; 0.40–0.59, “moderate”; 0.60–0.79, “strong; and 0.80–1.00, “very strong” (60). Only correlations with *P* ≤ 0.05 were considered statistically significant.

## 3 Results

### 3.1 Characterizing animal health status

All heifers had a median CRS of 0 and a median fecal score of 0 at all clinical assessment points from birth to 8 mo (available on OSF). At weaning (d90 post-birth), seven healthy calves had a TUS score of 0 and three had a TUS score of 1. In the subclinical cohort, five calves had TUS scores of 2b and five had scores of 3 (Table 2). At 8 mo (d244 post-birth), all heifer calves had median CRS of 0 and a median fecal score of 0. Regarding TUS, six healthy heifers had TUS scores of 0 and four had scores of 1. Of the ten heifers that were determined to have subclinical BRD at weaning, 80% continued to have some form of latent subclinical BRD based on their TUS scores at 8 mo.

**Table 2:** Clinical assessment and thoracic ultrasonography score frequencies across the sample population.

Clinical assessment and TUS score frequencies across sample pool								
	Healthy (HE) *n* = 10		Subclinical (SC) *n* = 10		Holstein-Friesian (HO) *n* = 14		Jersey (JE) *n* = 6	
	HO *n* = 7	JE *n* = 3	HO *n* = 7	JE *n* = 3	HE *n* = 7	SC *n* = 7	HE *n* = 3	JE *n* = 3
Score	W TUS	8 TUS	W TUS	8 TUS	W TUS	8 TUS	W TUS	8 TUS
0	7	6	0	0	5	4	2	2
1	3	4	0	2	2	5	1	1
2a	0	0	0	3	0	3	0	0
2b	0	0	5	1	4	1	1	0
3	0	0	5	4	3	1	2	3
Score	W CRS	8 CRS	W CRS	8 CRS	W CRS	8 CRS	W CRS	8 CRS
0	6	9	9	9	11	12	4	6
1	3	1	1	0	3	1	1	0
2	1	0	0	1	0	1	1	0
3	0	0	0	0	0	0	0	0
>3	0	0	0	0	0	0	0	0
**Score**	**W FAEC**	**8 FAEC**	**W FAEC**	**8 FAEC**	**W FAEC**	**8 FAEC**	**W FAEC**	**8 FAEC**
0	10	8	9	9	13	13	6	4
1	0	1	0	1	1	1	0	1
2	0	0	1	0	0	0	0	0
3	0	1	0	0	0	0	0	1

TUS, Thoracic ultrasonography, scores range from 0–5; where 0 indicates normal lung imagine and 2B or higher indicates the presence of lung lesions ≥ 1cm in size; W, Weaning time-point; 8, 8 mo time-point; CRS, Clinical Respiratory Score; where 0 indicates complete absence of clinical signs and scores ≥ 3 indicates observable signs of bovine respiratory disease; FAEC, fecal score at time of clinical assessment, ranging from 0–3, where 0 indicates complete absence of clinical signs of enteric disease and scores of ≥2 indicate presence of enteric disease; W, weaning time-point; 8, 8 mo time-point.

At birth, calves received colostrum of adequate quality, with a mean colostrum total IgG concentration of 93.65 mg/mL (SE 1.95; range: 48.98–129.39 mg/mL). There was no effect of health status or breed nor any interactions between the two, on serum total protein (STP) or serum total IgG (using sRID). Based on STP measures, two calves were determined to have poor PTI status, both were part of the subclinical cohort. However, serum total IgG (sRID) suggests that four heifers had poor PTI, one healthy and three subclinical. There were no effects of breed or health status on haptoglobin concentrations at either time-point, however, haptoglobin concentrations in all replacement heifers exceeded the normal range (0.0–0.05mg/mL; per assay manufacturer guidelines). While not significant, at weaning (d 90 post-birth; *P* > 0.10), healthy heifers had observably lower plasma haptoglobin concentrations than subclinical heifers (Table 3). At 8 mo, 12 heifers (six healthy and six subclinical) tested seropositive for anti-lungworm antibodies. Lungworm (*D. viviparus*) ELISA results influenced haptoglobin concentrations at 8 mo (d 244; *P* = 0.05), with heifers testing seronegative for lungworm antibodies showing higher haptoglobin concentrations (NEG: 1.3 mg/mL ± 0.24) compared to those testing seropositive (POS: 0.90 mg/mL ± 0.17).

**Table 3:** Immunological measurements of heifer calves with or without subclinical bovine respiratory disease at weaning and 8 months old.

Immunological data										
		All animals (*n* = 20)		Healthy (*n* = 10)		Subclinical (*n* = 10)		*P*		
	Unit	mean	SE	mean	SE	mean	SE	HS	Breed	Breed^*^HS
Colostrum IgG	mg/mL	93.65	1.95	87.91	4.08	98.57	3.86	NS	NS	NS
Serum total protein	g/dL	6.76	0.07	6.99	0.12	6.54	0.16	NS	NS	NS
Serum IgG	mg/mL	15.87	0.28	16.18	0.56	15.49	0.60	NS	NS	NS
Haptoglobin- weaning	mg/mL	0.35	0.01	0.28	0.02	0.35	0.01	NS	NS	NS
Haptoglobin- 8 months	mg/mL	1.08	0.04	1.14	0.09	1.02	0.04	NS	NS	NS
Lungworm ELISA	ODR	0.55	0.01	0.54	0.02	0.56	0.03	NS	NS	NS

SE, Standard Error; HS, Health Status; NS, Not significant; *P* > 0.05; IgG, Total Immunoglobulin G concentration; Lungworm ELISA, *D. viviparus* Enzyme-Linked Immunosorbent Assay, where ODR ≥ 0.50 indicates antibody seropositivity; ODR, Optical Density Ratio.

### 3.2 Evaluating animal performance overtime

At weaning, heifers were, on average, 90 d old (SE 0.31; range: 71–103 days old). There was no effect of breed or health status, nor any interaction, on age at weaning. There was an effect of breed on liveweights throughout the observational period (*P* < 0.05), where Holstein-Friesians were consistently heavier than Jersey heifers (Table 4). There was an effect of health status on all liveweights taken post-weaning to four months into gestation (*P* < 0.05). There were no interactions between breed and health status (P>0.10). At weaning, there was a tendency (*P* = 0.08) for healthy calves to weigh 7.6 kg (SE 3.13) more than subclinical calves (Table 4), by the fourth month of gestation healthy heifers weighed +55KG than subclinical heifers. Breed affected post-weaning average daily gain (ADG) (*P* < 0.001; from weaning to 8 mo), where Holstein-Friesians gained 280 grams per day more than Jerseys. Health status affected pre-weaning ADG (*P* = 0.04; birth to weaning) and post-weaning ADG (*P* = 0.02). Pre-weaning, healthy calves gained 90 grams more per day (SE 0.03) than their subclinical counterparts, this increased to 110 grams per day (SE 0.03) during the post-weaning period (weaning to 8 mo; additional data available on OSF).

**Table 4:** Mean heifer liveweights from birth to 4 months into first gestation.

Estimated marginal means of heifer live weights (kg) from pre-weaning to 4 months into gestation										
	*P*-value		HE-SC	Health Status			HO-JE	Breed		
	HS	Breed	Dif	Healthy	Subclinical	Pooled SE	Dif	Holstein	Jersey	Pooled SE
Weight- 3 weeks	NS	0.02	–	–	–	–	+7.5	45.7	38.2	2.00
Weaning weight	0.08	NS	+7.6	94.0	86.4	3.13	–	–	–	–
Post-weaning weight 1	0.04	0.0003	+14.0	125.0	111.0	4.42	+29.0	133.0	104.0	4.34
Post-weaning weight 2	0.04	0.0001	+16.0	131.0	115.0	4.71	+33.0	140.0	107.0	4.62
Weight- 8 months	0.004	0.00004	+31.0	197.0	166.0	6.40	+51.0	207.0	156.0	6.27
Post-service weight	0.03	0.001	+39.0	337.0	298.0	10.25	+57.0	346.0	289.0	10.07
Gestation weight	0.004	0.002	+55.0	381.0	326.0	11.30	+66.0	386.0	320.0	11.13

HS, Health Status; NS, Not Significant; *P* > 0.05; HE-SC, Healthy – Subclinical, where to determine the difference, the estimated marginal mean for subclinical heifers was subtracted from the estimated marginal mean of healthy heifers; Dif, difference between estimated marginal means; SE, Standard Error; HO-JE, Holstein-Friesian – Jersey, where to determine the difference, the estimated marginal mean for Jersey heifers was subtracted from the estimated marginal mean of Holstein-Friesian heifers; Post-service weight, weight taken after breeding via artificial insemination; Gestation weight, weight taken at 4th month of gestation.

### 3.3 Sequencing performance and overall composition

**Figure 2:**
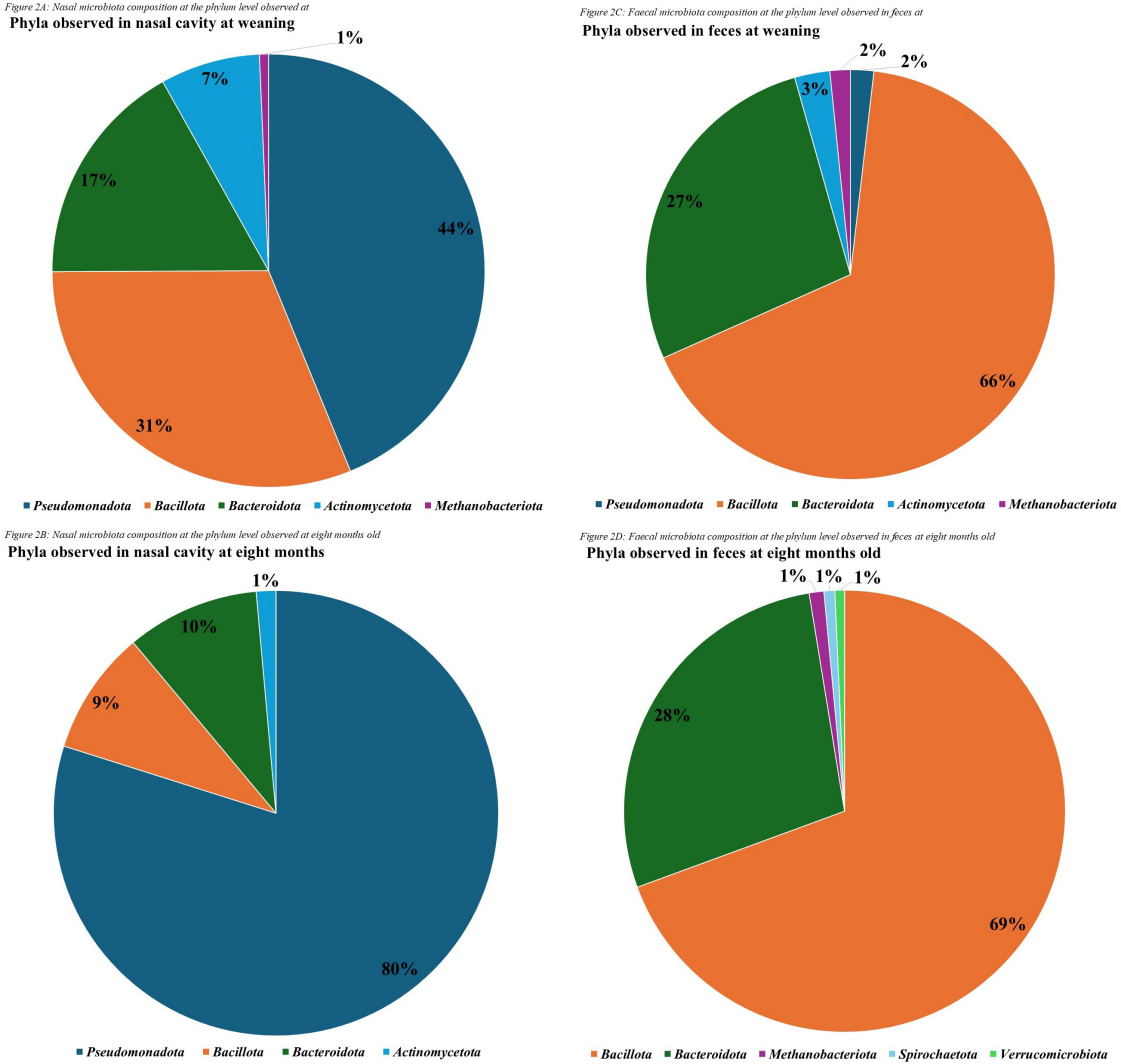
Primary phyla observed in the nasal cavity **(A; B)** and feces **(C; D)** of healthy and subclinical replacement heifers at weaning **(A; C)** and 8 mo **(B; D)**.

After quality filtering, merging and removal of chimeric sequences, an average of 76,810 (SE 2,630) reads per sample were generated, ranging from 19,663 to 107,450 reads per sample. Fecal samples had an average of 92,433 (SE 1,408) reads per sample, whereas nasal samples had an average of 61,988 (SE 3,597) reads per sample (Table 5). This resulted in 10,798 unique ASVs identified at the species level. Decontamination resulted in the removal of 1.36% of these ASVs. Post decontamination and after filtering for a RA of >0.01%, a total of 791 unique ASVs were identified across all four datasets, with 121 ASVs observed in both the nasal and fecal samples at weaning and 8 mo (available on OSF). Overall, the primary phyla observed across all four datasets, in various relative abundances, were: *Bacillota, Bacteroidota, Pseudomonadota, Actinomycetota* and (domain: archaea) *Methanobacteriota* (Figures 2A–D).

**Table 5:** Mean read counts generated per sample for all data (nasal and fecal samples at both time-points), and by sample type, by time-point, and by sample type at each time-point.

Mean read counts per sample post-processing					
Data	Mean	SE	Minimum	Maximum	ASVs
All data	76,810	2,630	19,663	107,450	791
Nasal samples	61,988	3,597	19,663	94,660	282
Fecal samples	92,433	1,408	69,053	107,450	630
Weaning samples	80,049	3,689	28,755	107,450	493
Eight-month samples	73,896	3,717	19,663	107,426	397
Nasal samples at weaning	68,629	5,544	28,755	94,660	245
Fecal samples at weaning	92,811	2,260	69,053	107,450	356
Nasal samples at 8 mo	55,680	4,301	19,663	83,864	61
Fecal samples at 8 mo	92,112	1,811	76,111	107,426	359

SE, standard error; ASVs, total number of unique amplicon sequence variants observed post-filtering for a relative abundance of >0.01%.

Positive controls run for each kit were very strongly correlated to one another with *r* ranging from 0.93–0.98 (*P* < 0.001). ANOVA revealed that there was an effect of time on α-diversity (α; Shannon) for nasal and fecal samples (*P* < 0.0001), but no effect of breed (*P* > 0.10) or health status (*P* > 0.10). Based on PERMANOVA, a sample × time-point interaction was observed (*P* = 0.001), additionally, sample type (Fecal or Nasal; *P* = 0.0001) and time-point (weaning or 8 mo; *P* = 0.0001) had significant effects on beta-diversity (β; composition). During assessment of homogeneity of group dispersions, composition differed significantly by sample type (*P* < 0.01). When separated by sample type, fecal samples were observed to be significantly different in composition based on time-point (*P* < 0.0001), nasal samples were observed to be homogenous across sample groups (*P* > 0.10). PERMANOVA by sample type indicated an effect of time-point (*P* = 0.001) on β-diversity. When separated by sample and time-point, health status influenced the β-diversity of the nasal microbiota at weaning (*P* = 0.01) and showed a tendency to affect fecal microbiota at weaning (*P* = 0.08; data available on OSF). Breed had no effect on any sequencing data, either when analyzed as an overall data set or when stratified by sample type and time-point. Similarly, health status had no effect on nasal or fecal microbiota at 8 mo, and lungworm ELISA status showed no effect on nasal or fecal microbiota composition.

### 3.4 Temporal dynamics and general composition

Differential abundance analysis revealed that 264 nasal ASVs and 555 fecal ASVs were significantly associated with time-point (adj.p ≤ 0.05; data available on OSF).

#### 3.4.1 Nasal microbiota

**Figure 3:**
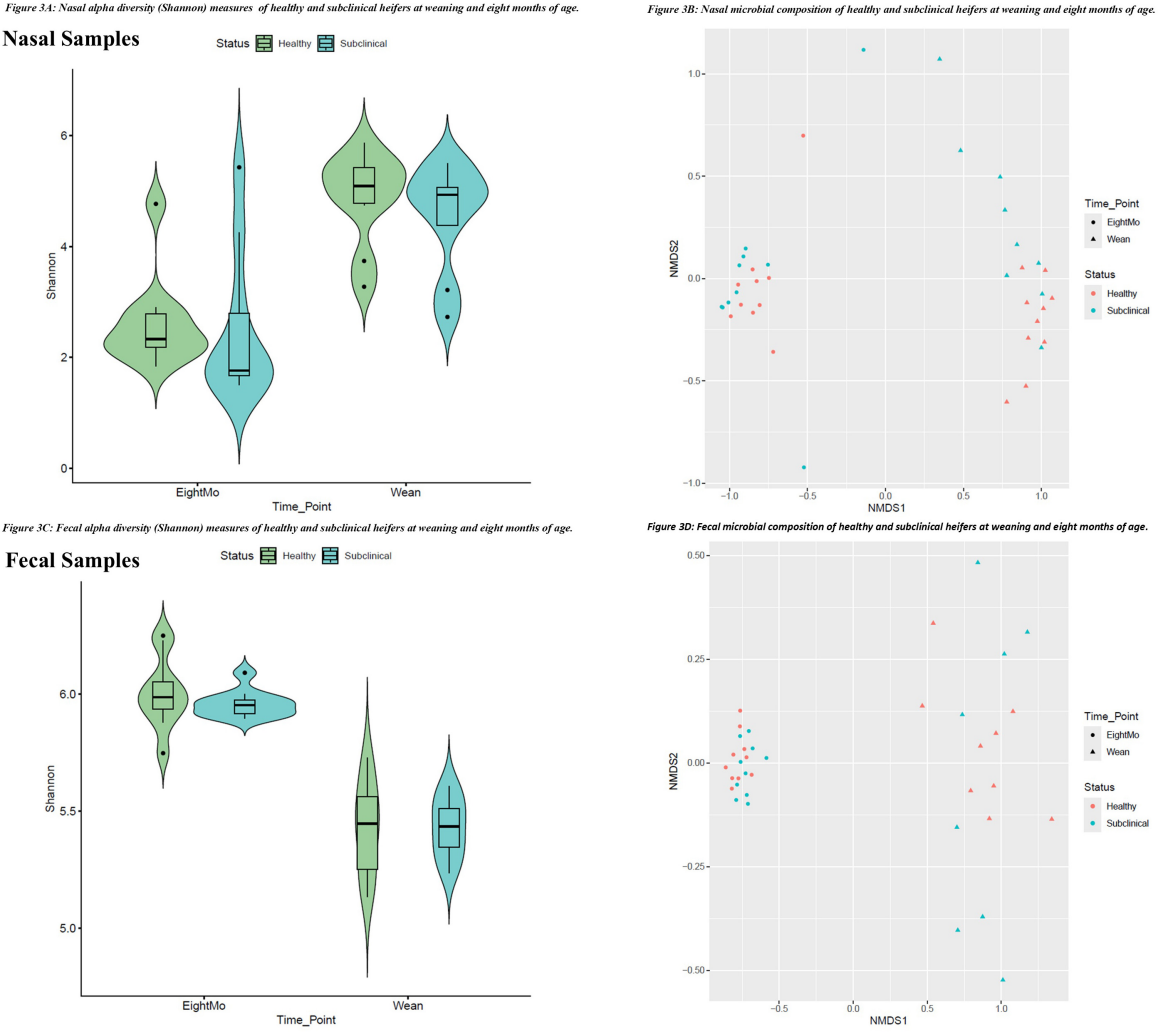
Alpha (α) diversity of the nasal **(3A)** and fecal **(3C)** microbiota of healthy and subclinical heifers at 8 mo and at weaning and beta (β) diversity depicting microbial community composition through the use of non-metric multidimensional scaling (NMDS) Bray-Curtis plots for the nasal cavity **(3B)** and feces **(3D)** at weaning and 8 mo for heifers with or without subclinical bovine respiratory disease (BRD).

The nasal microbiota at weaning was observed to be greater in α-diversity, with a mean Shannon Index score of 4.74 (SE 0.23; Figure 3A), but had more heterogeneity in community membership across samples (Figure 3B), than the nasal microbiota at 8 mo [mean α-diversity: 2.55 (SE 0.22; *P* < 0.0001)]. Time-point also had an effect on dominance and rarity measures. The nasal microbiota at 8 mo saw a decrease in ASVs determined to contribute to core abundance [−1.44 percentage points (pp); *P* < 0.05]. Additionally, ASVs identified as contributing to rare abundance decreased significantly from weaning to 8 mo (−25pp; *P* < 0.0001), a similar decrease was observed in low abundance ASVs from weaning to 8 mo (−17pp; *P* < 0.001) (data available on OSF).

At weaning, *Moraxella bovis* (ASV 5; RA = 9.31%), *Moraxella bovoculi* (ASV 6; RA = 4.77%), *Pasteurella multocida* (ASV 3; RA = 4.22%), *Acinetobacter lwoffi* (ASV 13; RA = 3.61%) and *Filobacterium* sp. (ASV 17; RA = 3.53%) were the five most proportionally abundant ASVs observed in the nasal microbial community. At 8 mo the top five most proportionally abundant ASVs were: *f_Moraxellaceae* unclassified genus (ASV 1; RA = 39.02%), *Mannheimia* sp. (ASV 2; RA = 13.42%), *Pasteurella multocida* (ASV 3; RA = 7.86%), *Moraxella bovoculi* (ASV 6; RA = 4.93%), and *Filobacterium* sp. (ASV 8; RA = 4.75%). Additionally, at weaning there were six ASVs within the domain archaea, taxonomically identified as *Methanobrevibacter spp*. (ASVs 10,754–10,756, 10758; total RA = 0.39%) and *Methanosphaera spp*. (ASVs 10,757, 10,760; total RA = 0.14%), however, these archaea were no longer present in the nasal microbiota at 8 mo.

#### 3.4.2 Fecal microbiota

The fecal microbiota at weaning was observed to have reduced α-diversity, with a mean Shannon Index score of 5.43 (SE 0.03; Figure 3C), and greater heterogeneity across samples (Figure 3D), than the fecal microbiota at 8 mo (mean α-diversity: 5.98 (SE 0.03; *P* < 0.0001)). Time-point also had an effect on dominance and rarity measures. The fecal microbiota at 8 mo saw an increase in ASVs determined to contribute to core abundance (+7pp; *P* < 0.0001). Whereas ASVs identified to contribute to rare abundance decreased from weaning to 8 mo (−11.6pp; *P* < 0.0001), while low abundance ASVs increased from weaning to 8 mo (+16pp; *P* < 0.001; data available on OSF).

At weaning, four of the 10 most proportionally abundant ASVs were identified as *f_Oscillospiraceae UCG-005 spp*. (ASVs 4, 7, 11, 18; total RA = 7.47%). Two ASVs identified as *f_Rikenellaceae RC9 gut group spp*. (ASV 25, 40; total RA = 2.28%), *Leyella* sp. (ASV 16; RA = 1.90%), *Bifidobacterium pseudolongum* (ASV 20; RA = 1.41%), *f_Muribaculaceae* unclassified genus (ASV 28; RA = 1.35%) and *Bacteroides* sp. (ASV 34; RA = 0.89%) were also present in the top 10 ASVs observed in feces at weaning. Five ASVs belonging to the domain archaea, identified as *Methanobrevibacter spp*. (ASV 10,754–10,756, 10,758, 10,759; total RA = 0.91%) and three *Methanosphaera spp*. (ASV 10,757, 10,760, 10,761; total RA = 0.33%) were present at weaning. At 8 mo, five of the 10 most proportionally abundant ASVs belonged to *f_Oscillospiraceae UCG-005 spp*. (ASV 4, 7, 11, 12, 21; total RA =11.44%). Two ASVs identified as *Alistipes spp*. (ASV 14, 33; total RA = 3.32%), *Paeniclostridium* sp. (ASV 10; RA = 2.95%) and *Romboutsia ilealis* (ASV 9; RA = 2.64%) also contributed to the 10 most proportionally abundant ASVs in feces at 8 mo. Four ASVs identified as *Methanobrevibacter spp*. (ASV 10,754–10,756, 10,758; total RA = 0.72%), one *Methanosphaera* sp. (ASV 10,757; RA = 0.05%) and one *Methanocorpusculum* sp. (ASV 10,762; RA = 0.10%) were also observed in feces at 8 mo.

### 3.5 Microbiota associated with health status

A total of 40 ASVs were determined to be associated with heifer BRD status (adj.*p* ≤ 0.10). Nine nasal ASVs had an adj.*p* of ≤ 0.05, seven of these were only present at weaning, one was only present at 8 mo, and one was present at both time-points. One of these ASVs was significantly associated with health status only, all others were also significantly associated with time. Twenty-four fecal ASVs had an adj.*p* ≤ 0.05; 12 of these were only observed at weaning, five were only observed at 8 mo and two were present across both time-points. Again, one ASV was significantly associated with health status only, all other significant fecal ASVs were also significantly associated with time (Table 6).

**Table 6:** ASVs significantly associated with BRD-status in the nasal cavity and feces of replacement heifers.

ASVs significantly associated with heifer BRD-status							
	ASV	adj.p	Microbe	RA at weaning		RA at 8 mo	
				HE	SC	HE	SC
Nasal ASVs	56	0.01	*Oscillospiraceae UCG-005* sp.	0.13%	0.04%	–	–
	867	0.02	*Xylanibacter* sp.	0.03%	0.17%	–	–
	37	0.02	*Bacteroides vulgatus*	0.33%	0.20%	–	–
	50	0.03	*Pseudomonas caeni*	1.30%	0.73%	–	–
	986	0.03	*Dietzia maris*	–	–	0.10%	0.04%
	215	0.04	*Bacteroides fluxus*	0.08%	0.05%	–	–
	9^*^	0.05	Romboutsia ilealis^*^	0.61%	0.45%	0.53%	0.42%
	34	0.05	*Bacteroides* sp.	0.52%	0.33%	–	–
	451	0.05	*Erysipelotrichaceae UCG-002 bacterium*	0.10%	0.07%	–	–
Fecal ASVs	174	0.004	*Bacteroides* sp.	0.00%	0.56%	–	–
	547	0.006	*Ruminococcus* sp.	–	–	0.04%	0.13%
	570	0.006	*Rikenellaceae RC9 gut group* sp.	–	–	0.10%	0.01%
	695	0.01	*Bacteroidales RF16 group unclassified genus*	–	–	0.11%	0.01%
	263	0.01	*Blautia glucerasea*	0.24%	0.10%	–	–
	121	0.01	*Rikenellaceae RC9 gut group* sp.	0.00%	0.85%	–	–
	88	0.02	*Blautia* sp.	0.56%	0.22%	–	–
	234	0.02	*Bacteroides* sp.	0.25%	0.09%	–	–
	584	0.02	*Marvinbryantia* sp.	0.10%	0.04%	–	–
	51	0.03	*Phascolarctobacterium* sp.	0.81%	0.40%	–	–
	711	0.03	*o_Clostridia vadinBB60 group unclassified family*	0.03%	0.10%	–	–
	392	0.03	*Rikenellaceae RC9 gut group* sp.	0.00%	0.28%	–	–
	59	0.03	*Oscillospiraceae UCG-005* sp.	0.08%	0.33%	0.41%	0.53%
	168^*^	0.04	Butyricioccaceae UCG-009 sp.^*^	0.08%	0.22%	0.13%	0.16%
	295	0.04	*Phascolarctobacterium* sp.	0.00%	0.43%	–	–
	247	0.05	*Parasutterella secunda*	0.23%	0.10%	–	–
	379	0.05	*o_Bacteriodales RF16 group unclassified genus*	–	–	0.24%	0.01%
	579	0.05	*Holdemanella* sp.	0.06%	0.07%	–	–
	743	0.05	*Lachnospiraceae unclassified genus*	–	–	0.03%	0.07%

ASV, Amplicon sequence variant; adj.p, q value determined during correction for false discovery via Benjamini-Hochberg procedure; RA, Relative abundance; HE, Healthy heifer cohort; SC, Subclinical BRD heifer cohort; ^*^ASV came back as significantly associated with health status only. No significant association with time.

#### 3.5.1 Nasal microbiota

**Figure 4:**
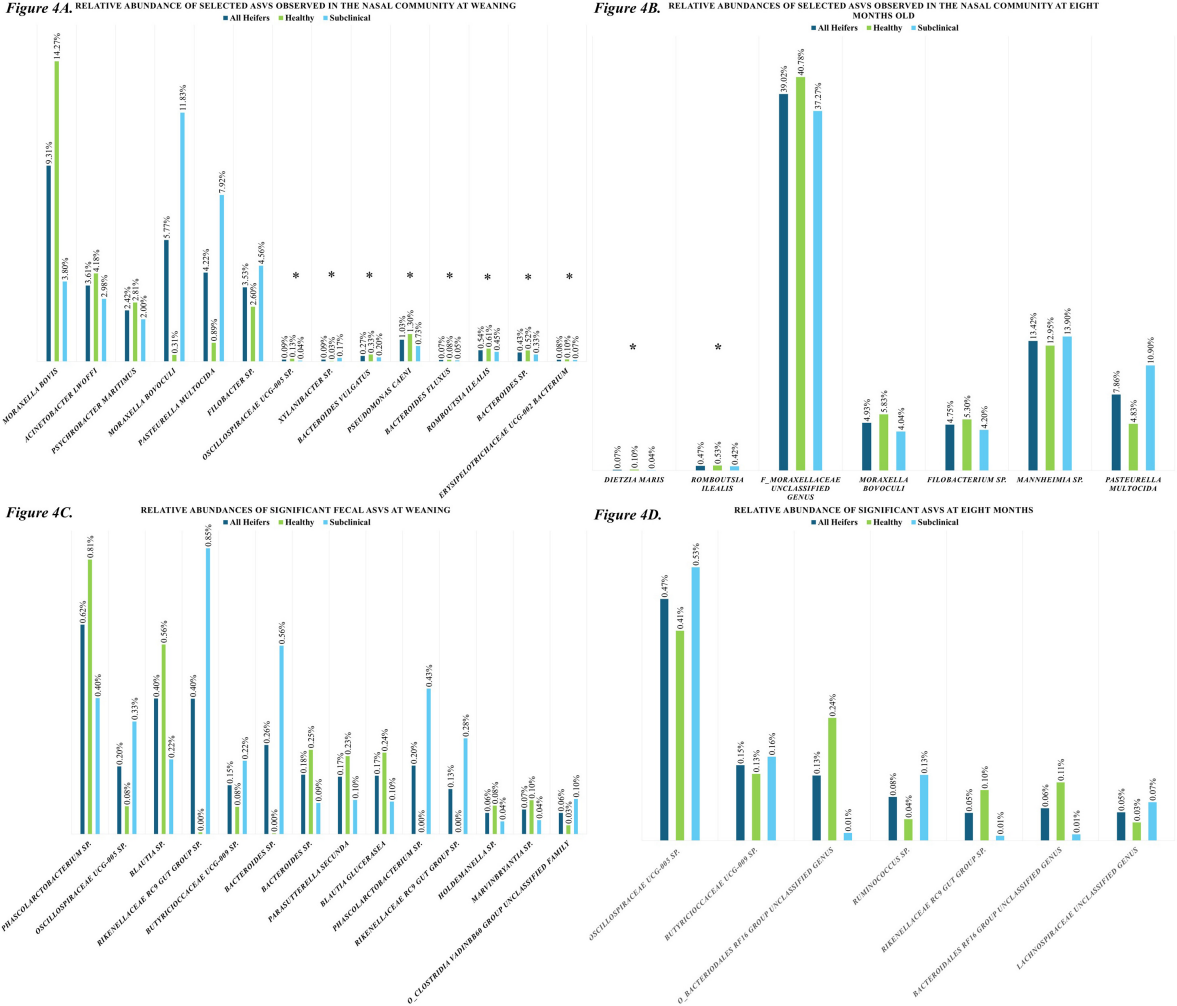
Relative abundances of **(A)** selected Nasal ASVs observed at weaning; **(B)** selected Nasal ASVs observed at 8 mo.; **(C)** Fecal ASVs determined to be significantly associated with BRD-status at weaning; and **(D)** Fecal ASVs determined to be significantly associated with BRD-status at 8 mo. In figures **(4A)** and **(4B)**, a * denotes an adj.*p* value of ≤ 0.05. All ASVs depicted in **(4C)** and **(4D)** have an adj.*p* value of ≤ 0.05.

Healthy heifers had slightly greater Shannon index scores than subclinical heifers (α-diversity; HE = 4.90 (4.25–5.55), SC = 4.41 (3.74–5.08), pooled SE = 0.31; *P* > 0.10) at weaning. This difference did not continue on to 8 mo (HE = 2.60 (1.77–3.43), SC = 2.67 (1.84–3.50), pooled SE = 0.39; *P* > 0.10; Figure 3A). Microbial composition (β-diversity), visualized using NMDS Bray-Curtis plots (Figure 3B), shows that subclinical heifers group together slightly at both timepoints. At weaning, they were more heterogenous in composition than healthy heifers. There were observable differences in the phyla observed in the nasal microbiota at weaning, where *Bacillota* (+3.53pp) and *Actinomycetota* (+2.31pp) were enriched in healthy heifers and *Bacteroidota* (+3.40pp) *Pseudomonadota* (+4.38pp) were enriched in subclinical heifers. At the species level, the nasal microbiota of healthy heifers at weaning was dominated by *Moraxella bovis* (ASV 5; RA = 14.27%)*, Acinetobacter lwoffii* (ASV 13; RA = 4.18%) and *Psychrobacter maritimus* (ASV 17; RA = 2.81%). While not found to be significant, these ASVs were observably greater in healthy heifers than the subclinical cohort (ASV5: = +10.47pp; ASV 13: +1.20pp; ASV 17: +0.80pp). In the subclinical cohort, *Moraxella bovoculi* (ASV 6; RA = 11.83%)*, Pasteurella multocida* (ASV 3; RA = 7.92%) and *Filobacterium* sp. (ASV 8; RA = 4.56%) were proportionally the most abundant. Similarly, these ASVs were observably higher in subclinical heifers than in the healthy cohort (Figure 4A; ASV 6: +11.52pp; ASV 3: +7.03pp; ASV 8: +1.95pp). At 8 mo, the nasal microbiota of healthy heifers was dominated by an unclassified genus in the *Moraxellaceae* family (ASV 1; RA = 40.78%), *Mannheimia* sp. (ASV 2; RA = 12.95%), *Moraxella bovoculi* (ASV 6; RA = 5.83%), *Filobacterium* sp. (ASV 8; RA = 5.30%), and *Pasteurella multocida* (ASV 3; RA = 4.83%). All five of these also dominated the nasal microbiota of subclinical heifers at 8 mo, however, there were observable differences in relative abundances, particularly *Pasteurella multocida* (ASV 3; +6.07pp) which was proportionally greater in subclinical animals and *f_Moraxellaceae* (ASV 1; +3.51pp) which was proportionally greater in healthy heifers (Figure 4B; additional data available on OSF). However, none of these ASVs were significantly associated with BRD status.

The ASVs found to be significantly associated with heifer BRD-status, were lowly abundant community members in both cohorts at both timepoints (adj.*p* ≤ 0.05; Table 6). At weaning, seven of the significant ASVs were proportionally greater in healthy heifers (Figure 4A). Both ASVs present at 8 mo were proportionally greater in healthy heifers (Figure 4B). *Romboutsia ilealis* (ASV 9) was the only ASV observed in the nasal microbiota that was significantly associated with BRD-status only and was proportionally greater in healthy heifers at weaning and 8 mo. At weaning, ASV 10760, identified as *Methanosphaera* sp., tended to be proportionally greater in the nasal cavity of healthy heifers than subclinical heifers (adj.*p* = 0.06; HE: RA = 0.08%, SC: RA = 0.03%).

#### 3.5.2 Fecal microbiota

Shannon index scores between healthy and subclinical heifers did not differ greatly (HE = 5.41 (5.28–5.54), SC = 5.40 (3.74–5.08), pooled SE = 0.06; *P* = 0.08) at weaning or at 8 mo (HE = 5.97 (5.89–6.04), SC = 5.96 (5.89–6.03), pooled SE = 0.03; *P* > 0.10; Figure 3C). Microbial composition (β-diversity), visualized using NMDS Bray-Curtis plots (Figure 3D), showed that healthy heifers appeared to be more homogenous than the subclinical cohort at weaning, with little difference at 8 mo. At weaning the predominant species level ASVs identified did not differ greatly between cohorts (data available on OSF). However, there were observable differences at the phylum level. At weaning, the fecal microbiota of healthy heifers saw greater abundances of *Bacillota* (+3.88pp) and *Pseudomonadota* (+1.21pp), whereas *Bacteriodota* (+3.63pp) and *Actinomycetota* (+1.31pp) were enriched in the subclinical cohort (data available on OSF). *Romboutsia ilealis* (ASV 9) was one of the 10 most proportionally abundant ASVs in healthy heifers and was greater than it was in the subclinical cohort (HE: RA = 1.26%, SC: RA = 0.46%), but this difference was not significant in feces (adj.p >0.10). During differential abundance analysis, a total of 14 ASVs present at weaning were identified as significantly associated with BRD-status (adj.*p* ≤ 0.05; Figure 4C). At 8 mo, the predominant microorganisms within the community did not differ greatly between cohorts (data available on OSF). Differential abundance analysis suggests that seven ASVs at 8 mo were significantly associated with BRD-status (Figure 4D). One ASV, *f_Butyricioccaceae UCG-009* sp. (ASV 168; adj.*p* = 0.04), was significantly associated with BRD-status only, and was proportionally greater in subclinical heifers at both time-points.

### 3.6 Associations between health-associated microorganisms and animal measurements

**Figure 5:**
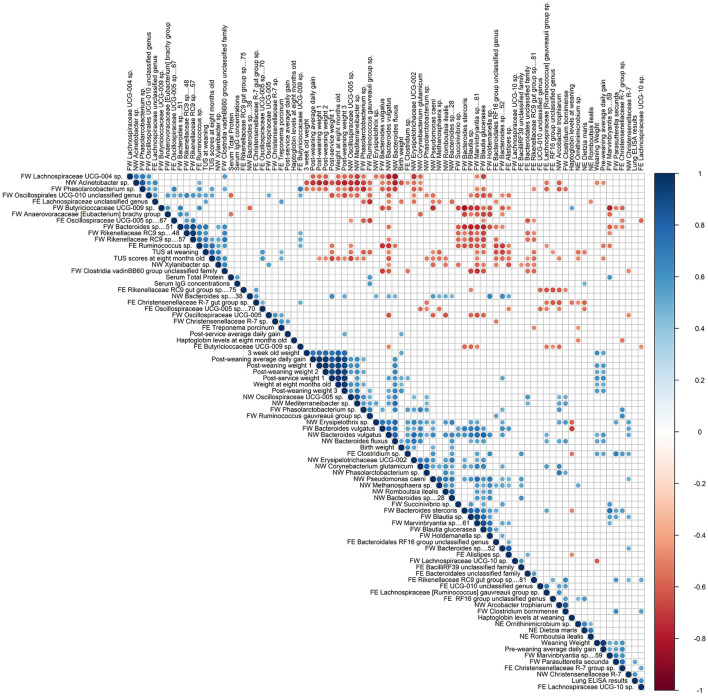
Spearman's rank order correlations between heifer performance, immunological measures, TUS scores, and ASVs associated with BRD-Status.

Thirty-one very strong, 193 strong and 456 moderate correlations were observed between heifer performance measures, immunological measures, TUS scores and ASVs associated with BRD-Status (*P* ≤ 0.10; Figure 5). Serum total protein measures were moderately correlated to serum IgG concentration (r_s_ = 0.54; *P* = 0.01). All weights and ADGs were moderately to very strongly correlated to one another (data available on OSF). Thoracic ultrasonography scores were strongly positively correlated to each other (r_s_ = 0.78; *P* < 0.0001). Weaning TUS scores were strongly correlated to ASVs observed in the nose and feces at weaning and 8 mo [Weaning: Nasal ASV 867 (r_s_ = 0.65); Fecal ASVs: 174 (r_s_ = 0.72), 392 (r_s_ = 0.67), 234 (r_s_ = −0.66); 8 mo: Fecal ASV 547 (r_s_ = 0.70) *P* ≤ 0.001)]. Weaning TUS scores were also moderately correlated to gestation weight (4th month of gestation; r_s_ = −0.54, *P* = 0.02). TUS scores at 8 mo were strongly correlated to gestation weight (r_s_ = −0.66, *P* = 0.003) and moderately correlated to other weights (data available on OSF). Eight-month TUS scores were also strongly correlated (P ≤ 0.01) to three weaning nasal ASVs [867 (r_s_ = 0.69), 56 (r_s_ = −0.67), 37 (r_s_ = −0.62)], one weaning fecal ASV [711 (r_s_ = 0.61)] and one fecal ASV at 8 mo 547 (r_s_ = 0.74). Haptoglobin levels at weaning were strongly correlated to weaning fecal ASV 37 (r_s_ = −0.70, *P* = 0.002).

At weaning, several nasal ASVs were associated with growth-related variables. ASV 648 (*Acinetobacter* sp.; BRD-status adj.*p* = 0.06) saw a variety of strong and moderate correlations with liveweights and both pre- and post-weaning ADG (r_s_= −0.47 to −0.77; *P* ≤ 0.04). Another nasal ASV, ASV 215 (*Bacteroides fluxus;* BRD-status adj.*p* = 0.04), demonstrated strong correlations with post-weaning liveweight measurements (r_s_ = 0.67, 0.66; *P* = 0.002) and post-service weight (r_s_ = 0.65; *P* = 0.003), and moderate correlations with pre- and post-weaning ADG, 8 mo weight, and gestation weight (r_s_ = 0.54–0.59; *P* ≤ 0.02). In fecal samples at 8 mo, ASV 641 showed a strong correlation with pre-weaning ADG (r_s_ = 0.62, *P* = 0.005). Numerous additional correlations were observed between nasal and fecal ASVs across both sampling points (data available on OSF). These associations should be interpreted with caution. Correlation analyses in a small cohort, particularly involving low abundance taxa, are exploratory and may not reflect biologically meaningful relationships.

## 4 Discussion

Twenty heifers were retrospectively classified at weaning into two groups – healthy or subclinical BRD – based on clinical assessments and TUS findings. Heifers, regardless of health status, showed no observable signs of respiratory disease at any assessment point, resulting in the classification of “subclinical” BRD. Immunological measurements were not significantly associated with BRD-status, although there were observable differences between cohorts. Lung lesions ≥ 1 cm in diameter were detected in the subclinical cohort at weaning. The majority of these heifers continued to have subclinical respiratory issues at 8 mo (five months after initial detection and pasture turn-out). Additionally, there were observable differences between healthy and subclinical heifer liveweights and average daily gains. PERMANOVA revealed that BRD-status had a significant effect on overall composition of the nasal microbiota and a tendency to effect the fecal microbiota at weaning. This effect was not observed at 8 mo. A variety of associations were observed between performance measures, TUS scores and ASVs associated with BRD-status indicating the potential for long-term systemic interplay between disease, the microbiota and animal growth. Further study, using larger cohorts and longitudinal sampling is required to confirm these findings.

### 4.1 Subclinical BRD may alter replacement heifer phenotypes

Subclinical BRD appeared to have a lasting impact on replacement heifer development, independent of PTI. In this study, calves received high-quality colostrum, with mean IgG concentrations of 93.65 mg/mL. Serum total protein values indicate that calves had excellent PTI (excellent ≥6.2 g/dL; mean STP: 6.76 g/dL). However, based on serum IgG concentrations determined using sRID assays, heifers shifted from excellent to fair PTI (fair :10 – 17.9 mg/mL; mean serum IgG: 15.87 mg/mL). Serum total protein is an indirect measure of IgG, and as such is only an estimation, which should be interpreted cautiously when determining calf PTI (61). Thompson and Smith (62) further note that both STP and sRID may lack precision under certain conditions, reinforcing the need for careful interpretation and complementary diagnostic approaches. Although, sRID remains the gold standard for IgG quantification, it is not without limitations, including cost, time requirements, and inter-assay variability (63). The moderate correlation (r_s_ = 0.54; *p* = 0.01) between STP and serum IgG suggests that when used appropriately, STP is still an adequate proxy for evaluating serum IgG on farm. Importantly, the manifestation of subclinical BRD in this cohort did not appear to be directly linked to PTI status. This same finding is further supported by Scully et al. (40), who found that pre-weaning diarrhea occurred independently of PTI status in calves from the same research farm, highlighting the complex interplay between immunity, microbiota, and disease susceptibility.

From birth to 8 mo, no clinical signs of respiratory or enteric disease were observed during clinical assessment. TUS enabled the detection of subclinical BRD at weaning; however, it likely started to develop earlier in the pre-weaning period. Donlon et al. (18) investigated the performance of clinical scoring systems alongside the use of TUS and found that clinical BRD diagnosis depended greatly on the interpretation of the results. Normally, CRS ≥ 5 is indicative of clinical BRD (16, 21), however, in the present study a more aggressive threshold of > 3 was implemented to ensure true “healthy” and “subclinical” classifications. Even with this more aggressive cut off the clinical assessment and CRS of subclinical heifers at birth, d7, d21 and weaning were not indicative of BRD. These heifers continued to present as asymptomatic at all post-weaning time points, even though at 8 mo TUS was indicative of ongoing impairment of the lungs. This highlights the serious limitations of clinical assessment in early detection of BRD. Clinical assessment alone does not allow for the detection of subclinical disease, the precursor to clinical observable disease.

Haptoglobin concentrations at weaning were above the normal threshold (>0.05 mg/mL), which is likely associated with the stress of weaning. Elevated plasma haptoglobin has previously been associated with confirmed BRD cases in pre-weaned calves (64). Although BRD-status had no statistically significant effect on haptoglobin concentrations at weaning, subclinical heifer haptoglobin concentrations were slightly higher than the healthy cohort. This observation suggests that the presence of subclinical BRD may have exacerbated the stress experienced by heifers at weaning. Five months after weaning and initial pasture turnout (8 mo), the majority of subclinical heifers continued to have subclinical respiratory issues, from comet tails to lobar pneumonia, supporting the assertion that pre-weaning disease manifestation puts the animal at higher risk of disease manifestation later in life.

Lungworm (*D. viviparus*) infections are common in pasture-based systems that rely on grazing. Prevalence in Ireland has been reported at approximately 63% (65). In the present study, the second clinical assessment, TUS scoring and sample collection occurred in autumn five months after introduction to pastures. *Dictyocaulus viviparus*-specific ELISA antibody detection was performed to ensure potential lungworm infection was considered during analysis. As this was the first time heifers were at pasture, positive results are indicative of exposure during their first grazing season. Twelve heifers tested seropositive for lungworms; of these, six were classified as having subclinical BRD and six were healthy. Lungworm antibody results were not statistically associated with BRD-status at weaning (subclinical or healthy). It was, however, significantly associated with plasma haptoglobin concentrations at 8 mo, where animals that had negative ELISA results, had higher plasma haptoglobin. This suggests that seronegative heifers may have had an early-stage parasitic infection, as antibodies take approximately 28–44 days post infection to present in blood circulation (47, 66). This relationship between lungworm exposure and inflammation (haptoglobin concentrations) was unexpected. It is possible that this could be related to immune response timing, where seropositive heifers may have already mounted and resolved their immune response, leading to lower haptoglobin levels by the time of sample collection. It is also possible that seronegative heifers may have had recent exposure to lungworms not yet reflected in antibody levels. Immune response to lungworm is much quicker than other helminthic infections (67). Eosinophilia is a primary feature of the bovine immune response to lungworm infection (67). These immune cells are a part of the innate immune response (68) and can be a source of pro-inflammatory cytokines (69). Haptoglobin is strongly associated with proinflammatory diseases (70), thus high levels of eosinophils associated with early-stage infection may explain higher haptoglobin levels of seronegative heifers. The herd was dosed with anthelminthics in August and September immediately prior to sampling; therefore, it is likely that anthelminthic administration prior to autumn, when clinical lungworm infections typically occur, reduced the severity of infection. No lungworm-associated clinical signs (dramatic weight loss, difficulty breathing, cough) were reported during the grazing season, further supporting the assertion that lungworm infection was not severe. Lungworm ELISA measurements and lungworm status (seropositive or seronegative) were found to have no effects on any other data collected for this study, including all liveweights, TUS scores at 8 mo, or sequencing data. To the authors knowledge, this is the first study to have performed TUS examinations on cattle with lungworm infection. Heifers that tested seropositive for lungworm had TUS scores at 8 mo ranging from zero to three, indicating that TUS may not be appropriate for investigating parasitic respiratory infections. It is possible at 8 mo, the subclinical BRD in lungworm positive heifers with higher TUS scores was exacerbated by the presence of lungworm (5). Subclinical heifers did not see drastic changes in their TUS scores from one timepoint to another, suggesting that lungworm infection is incidental, and the lesions observed in the subclinical cohort may be associated with unresolved consolidation at weaning.

In this retrospective observational study, liveweights from birth to first gestation and pre- and post-weaning ADG were examined to explore associations between subclinical BRD status at weaning and subsequent growth patterns. Heifers classified as subclinical had lower ADG during the pre-weaning period, consistent with reports from other studies that identified reduced growth in calves with lung lesions or respiratory disease indicators (3, 21, 71, 72). Conversely, Cuevas-Gómez et al. (15) observed no difference in ADG between calves with or without clinical BRD, although calves with severe lung lesions demonstrated reduced growth. In the present study, the divergence in ADG between groups increased during the post-weaning period (weaning to 8 mo), with healthy heifers gaining more per day than those classified as subclinical. This pattern was reflected in liveweight measurements across time, with healthy heifers remaining heavier and the difference between groups increasing as animal approached early gestation. While these results indicate an association between subclinical BRD at weaning and subsequent growth trajectories, the sample size and observational study design mean that these findings should be interpreted cautiously and cannot be used to infer causation.

### 4.2 Low and rare abundance microorganisms are key players in disease manifestation

Examining the temporal dynamics of the nasal and fecal microbiota post-weaning was beyond the scope of this study. Proper temporal dynamics analysis would require more than two time-points, and more frequent sampling. However, the significant reduction in α-diversity observed in the nasal microbiota between weaning and 8 mo warrants further investigation. A study by Lima et al. (29) found that the nasal microbiota of dairy calves from d3 to d35 post-birth was highly diverse, similar to what was observed at weaning in the present study. Another study by Lima et al. (25), found that colonization of the dairy calf URT begins immediately and progresses quickly during the first week of life. They also reported that between d3 and d35 post-birth, α-diversity was reduced at d14 compared to d3 and d35. Timsit et al. (73) examined the nasal microbiota of beef calves at weaning, at feedlot arrival and d40 after feedlot arrival. They observed that the nasal microbiota continued to change across all three time-points, indicating that the nasal microbiota is less stable than other microbial communities, making it at higher risk of dysbiosis. Unfortunately, most nasal microbiota studies in cattle have been conducted in feedlot or indoor housing systems (34), where BRD risk is higher and easier to monitor. Very few studies have specifically examined nasal microbiota dynamics in calves and cattle raised in pasture-based systems. To the authors knowledge this is the first study to report on the nasal microbiota of dairy heifer calves post-weaning and on pasture. The dramatic decrease in α-diversity may be associated with heifers living on pasture, but this is only speculative, as there is no research beyond the present study to support this statement. This observation highlights a serious requirement to investigate the temporal dynamics of the nasal microbiota in dairy cattle from birth to maturity in various types of production systems. It is also possible that lungworm infection may have altered the microbiota. There was no effect of lungworm antibody status on the nasal or fecal microbiota at 8 mo. However, it is possible that all heifers had lungworm infections at varying stages, making detection of microbial shifts difficult. A recent study of *Haemonchus contortus* in goats found that parasitic infection altered the abomasal microbiota, where bacterial load increased, archaea decreased and altered butyrate producing bacteria (74). The role of lungworms regarding changes observed in the nasal microbiota in the present study is unknown and any conclusions made are purely speculative. There is a paucity of literature on this topic. Future research should be holistic in nature and examine all components (prokaryotic, eukaryotic and viral) of the microbiota through the use of multi-omics (metagenomics, metatranscriptomics etc.) to understand better the impact of parasitic protozoa on the microbiome and the host.

In the present study, many of the ASVs identified belonged to those that are known as BRD pathogens: *Moraxella, Pasteurella, Mesomycoplasma* and *Mannheimia* (5–7). However, differential abundance analysis revealed that none of these were associated with heifer BRD-status. This agrees with Centeno-Martinez et al. (31) who found that the presence of BRD pathogens was not predictive of BRD manifestation and Centeno-Delphia et al. (32) who reported that these microorganisms were present in the nasal microbiota, regardless of disease status. The present study, alongside the aforementioned, supports the assertion that these microorganisms are normal members of the nasal microbiota with pathogenic potential.

All of the ASVs associated with BRD-status, in both the nasal and fecal microbiota, were low-abundance community members (RA < 1.5%). In a recent review article, Han and Vaishnava (35), detail the importance of low abundance microbiota in host-microbe interactions. Briefly, they point out that low abundance microorganisms are often removed from the dataset during initial filtering and are left out of later analyses. This is problematic because studies performed in humans and mice show that even lowly abundant bacteria and archaea significantly contribute to host immune development, disease and the overall microbial community. In the present study, the majority of these low abundance ASVs in the nose and feces were proportionally greater in healthy heifers. While not statistically significant, healthy heifers also had slightly higher α-diversity measures. This suggests that low abundance microorganisms may play an important role in the development of disease. Increased α-diversity measures in healthy calves have been previously reported in other BRD studies (28, 32, 75). This increased α-diversity combined with more prevalent low abundance microorganisms likely contribute to colonization resistance and stability, which could help prevent dysbiosis and pathogen proliferation. Additionally, this is the first study to report on the presence of archaea in the nasal microbiota, one of which tended to be present in greater abundance in healthy heifers, compared to subclinical ones. These findings highlight the potential importance of including low abundance community members in analysis. It also highlights the need to explore what role they may play in the development of BRD. Determining the exact cause of the microbial shift that resulted in disease and the pathogenic agent was beyond the scope of this study. However, it has clearly been depicted that increased α-diversity and greater proportions of low abundance microorganisms are key indicators of a healthy microbiome and heifer health. Future research should focus on longitudinal microbiota profiling, e.g., tracking the nasal microbiota composition from birth through post-weaning to identify when and how diversity declines. This work should be done using a multi-omics (metagenomics, metatranscriptomics, metabolomics etc.) approach paired with health records, animal performance and environmental data for a truly holistic understanding of disease and microbiomes.

Microorganisms and their by-products at the mucosal surface, in the respiratory and gastrointestinal tracts, interact with the host immune system and communicate with other host organs via crosstalk along the microbiome-gut-lung-axis (76, 77). While bidirectional, the majority of communication along this axis travels from the gut to the lung (77). Any disturbance to the microbiota at the mucosal surface directly results in alterations to host immune function which then impacts the crosstalk between microorganisms, the host immune system, and the other host organs along the axis (77). However, there is little research on this system in cattle and even less so on the impact of BRD on the gut microbiota, nor the role of the microbiome-gut-lung axis in association with BRD. The observed tendency of BRD-status to influence the overall composition of the fecal microbiota at weaning suggests that subclinical, moderate, respiratory infection may affect other microbial communities within the host. Yildiz et al. (78) reported that infection with Influenza A virus in mice induced gut microbiota dysbiosis and compromised mucosal barrier integrity. A similar study in mice also reported significant alterations to the gut microbiota following viral lung infection (79). Groves et al. (79) found that viral lung infection was associated with an increase in the phylum *Bacteriodota* and a decrease in *Bacillota*. Consistent with the aforementioned study, the present study found that at initial disease detection, subclinical calves exhibited an increase in *Bacteroidota* and a decrease in *Bacillota* within the fecal microbiota. A similar pattern was detected in the nasal microbiota of subclinical heifers at weaning. Given the paucity of research on the impact of BRD on the hindgut microbiota of cattle and calves, the authors can only speculate, based on the results, that the manifestation of subclinical BRD may influence the hindgut microbiota of heifers. The results highlight the need for more holistic, multi-omics approaches to understand the systemic consequences of disease manifestation.

### 4.3 The nasal and fecal microbiota associated with BRD-status may influence each other and the host

The wide range of associations reported in the present study may be indicative of systemic, longitudinal influences between microorganisms, microbiota sites, and the host itself. Early life weights were correlated to ASVs observed in the nose and feces at weaning and 8 mo. Nasal weaning microorganisms were strongly correlated to fecal ASVs at weaning and 8 mo. Weaning TUS scores were strongly correlated to TUS scores at 8 mo and fecal ASVs at weaning and 8 mo. All ASVs were those determined to be significantly associated with BRD-status, some of which were only present at the latter sampling time-point, five months after initial disease detection. Additionally, more fecal ASVs at weaning and 8 mo were significantly associated with BRD-status. This in combination with the many correlations observed suggests that subclinical BRD manifestation near weaning has long-lasting and systemic implications on replacement heifers. This is highlighted by the significant differences in weights and growth performance observed between the two cohorts. To the authors knowledge, this is the first study to report these kinds of relationships, and thus deeper insights into the mechanisms behind these associations would be purely speculative. Future research should take a systems-level approach to investigate how BRD influences the relationships between microbial communities at different mucosal sites and their collective impact on host physiology. Studies should aim to elucidate the mechanisms underpinning the microbiome–gut–lung axis, including the directionality and functional consequences of microbial and immune crosstalk between the gastrointestinal and respiratory tracts.

## 5 Limitations

This study employed a multi-dimensional approach — including repeated weights, TUS, haptoglobin assays, ELISA diagnostics, and microbiota sequencing — which provided data for each individual animal. Additionally, the homogeneity of the cohort in terms of age, management, and environmental exposure reduced confounding variability. Even so, the authors acknowledge that this study has certain limitations. Microbiome sampling occurred at discrete time points (weaning and 8 mo), which may have missed transient microbial shifts or immune responses. The lack of more frequent sampling limits ability to fully characterize microbiota dynamics and disease progression. Additionally, TUS does not detect parasitic infections like lungworms, and ELISA timing may have missed early-stage infections. It was not possible to conclusively determine whether heifers that tested seronegative for anti-lungworm antibodies were free of infection. The absence of clinically diagnosed BRD cases means the study focuses exclusively on subclinical disease, which, while valuable, restricts comparisons across the full spectrum of BRD severity. This limits the ability to draw conclusions about how microbial or physiological markers differ between subclinical and clinical BRD, or how disease progression might evolve from subclinical to clinical states. Although the sample size was limited (*n* = 20), it was powered appropriately for differential abundance analysis of healthy and subclinical microbial community composition. Future studies should aim to include more frequent sampling, larger cohorts and a broader range of BRD phenotypes to validate these findings and explore the continuum of disease manifestation.

## 6 Conclusions

This retrospective observational study showed that TUS identified lung lesions in heifers that exhibited no clinical signs of respiratory disease, highlighting the limitations of clinical scoring systems when used alone to evaluate respiratory health. Heifers classified with subclinical BRD at weaning displayed different growth patterns from healthy counterparts, although these associations should be interpreted cautiously. TUS findings also indicated that some respiratory abnormalities persisted beyond the pre-weaning period. Associations between BRD status and low abundance nasal and fecal microbiota were detected at both weaning and 8 mo, but these findings represent preliminary, exploratory signals and require validation in larger cohorts. Functional implications of these taxa cannot be determined from 16S rRNA gene amplicon sequencing alone. Overall, the study highlights the value of using multiple diagnostic approaches to characterize subclinical BRD under pasture-based management but does not allow for causal inference regarding growth performance or microbiota composition. Further research with larger, longitudinally sampled populations and functional microbial analyses is needed to clarify the relationships between respiratory health, microbial communities, immunity and performance in dairy heifers.

## Data Availability

The data set presented in this study can be found in online repositories. The names of the repository/repositories and accession number(s) can be found at: https://www.ncbi.nlm.nih.gov/, BioProject ID: PRJNA1359347. Additional supplementary material can be accessed through Open Science Framework at: https://osf.io/tnya9; DOI : 10.17605/OSF.IO/TNYA9.
